# Capillary Dynamics Regulate Post-Ischemic Muscle Damage and Regeneration in Experimental Hindlimb Ischemia

**DOI:** 10.3390/cells12162060

**Published:** 2023-08-14

**Authors:** Galina Wirth, Greta Juusola, Santeri Tarvainen, Johanna P. Laakkonen, Petra Korpisalo, Seppo Ylä-Herttuala

**Affiliations:** 1 Heart Center, Kuopio University Hospital, FI-70200 Kuopio, Finlandpetra.korpisalo@pshyvinvointialue.fi (P.K.); 2 A. I. Virtanen Institute for Molecular Sciences, University of Eastern Finland, FI-70211 Kuopio, Finland

**Keywords:** ischemia, hypoxia, capillary remodeling, muscle damage, regeneration, blood flow, hyperlipidemia

## Abstract

This study aimed to show the significance of capillary function in post-ischemic recovery from the perspective of physiological parameters, such as blood flow, hemoglobin oxygenation and tissue regeneration. Muscle-level microvascular alterations of blood flow and hemoglobin oxygenation, and post-ischemic myofiber and capillary responses were analyzed in aged, healthy C57Bl/6J mice (*n* = 48) and aged, hyperlipidemic LDLR^−/−^ApoB^100/100^ mice (*n* = 69) after the induction of acute hindlimb ischemia using contrast ultrasound, photoacoustic imaging and histological analyses, respectively. The capillary responses that led to successful post-ischemic muscle repair in C57Bl/6J mice included an early capillary dilation phase, preceding the return of arterial driving pressure, followed by an increase in capillary density that further supported satellite cell-induced muscle regeneration. Initial capillary enlargement was absent in the LDLR^−/−^ApoB^100/100^ mice with lifelong moderate hypercholesterolemia and led to an inability to recover arterial driving pressure, with a resulting increase in distal necrosis, chronic tissue damage and a delay in the overall recovery after ischemia. To conclude, this manuscript highlights, beyond arterial collateralization, the importance of the proper function of the capillary endothelium in post-ischemic recovery and displays how post-ischemic capillary dynamics associate beyond tissue blood flow to both hemoglobin oxygenation and tissue regeneration.

## 1. Introduction

The fundamental task of an organ-serving vasculature after an arterial occlusion is to re-establish tissue perfusion as soon as possible to minimize ischemic tissue damage. The central role of the collateral arterial network in restoring blood flow to ischemic skeletal muscle tissue has been well characterized [1,2]. Instead, the role of microvascular angiogenesis in this process has been considered almost negligible [1]. In general, upon an ischemic insult, a complex network of responses is activated at tissue level, initially by hypoxia and later by ischemia-induced tissue damage [3]. These responses promote angiogenesis and acute inflammation in the affected muscle [4,5], leading to increased capillary density. Also, the crosstalk between capillary endothelial and myogenic satellite cells during muscle regeneration further induces angiogenesis [6,7]. The presence of cardiovascular risk factors, such as increasing age and hypercholesterolemia, has been associated with an impaired arteriogenic/angiogenic regenerative capacity under ischemic conditions [8,9,10]. The tissue microvasculature is thus known to undergo remodeling in response to ischemia and likely does so for a purpose. How this remodeling is exactly related to the tissue recovery process is still not completely understood and may limit the translation of experimental pro-angiogenic therapies to the clinic.

In this study, the contribution of the microvasculature in post-ischemic blood flow and tissue recovery after acute hindlimb ischemia in aged, healthy C57Bl/6J mice and aged, hyperlipidemic LDLR^−/−^ApoB^100/100^ mice were investigated by using high-resolution contrast-enhanced ultrasound, state-of-the-art photoacoustic imaging, and histological analyses. This study demonstrates that ischemic muscle capillaries undergo dynamic changes, which are associated beyond muscle regeneration, with changes in blood flow parameters, microvascular hemoglobin oxygenation, as well as the severity and the duration of ischemic damage. These data display the importance of the microvasculature controlling skeletal muscle tissue fate after ischemia.

## 2. Materials and Methods

### 2.1. Experimental Animals

Healthy C57Bl/6J mice (*n* = 48, The Jackson Laboratory, Bar Harbor, ME, USA) and hyperlipidemic mice deficient of LDL receptor and expressing only apolipoprotein B100 (LDLR^−/−^ApoB^100/100^, C57Bl/6J background, (*n* = 69)), with a lifetime of a 3-fold increase in blood cholesterol levels and a humanized lipoprotein profile predisposing the animals to develop atherosclerosis even on a regular chow diet [11], were used. Notably, all mice were of old age (8–21 months), as advanced age is one of the most important risk factors for poor recovery and better represents the human population suffering from ischemic diseases. As metabolic parameters of healthy C57Bl/6J and hyperlipidemic LDLR^−/−^ApoB^100/100^ aged mice have been published by us previously, these analyses were not included in this study [12,13]. Mice were subjected to unilateral acute ischemia in the right hindlimb via permanent ligation of both the common femoral artery and vein proximal to the origin of the profound femoral artery branch. The femoral nerve was left undamaged. For the surgical procedure and imaging, the mice were anesthetized with isoflurane inhalation (induction: 4.5% isoflurane, 450 mL/min air; maintenance: 2.5% isoflurane, 250 mL/min air; Baxter International, Deerfield, IL, USA). Postoperatively, the mice were treated with analgesia (Rimadyl, 10 mg/kg, Pfizer, Dublin, Ireland). All experimental procedures for the animal studies described here were approved by the National Animal Experiment Board of Finland (license number: ESAVI/5343/04.10.07/2014) and carried out in accordance with the guidelines of The Finnish Act on Animal Experimentation. The animals were kept in standard housing conditions and fed a standard chow diet in the National Animal Laboratory Center of the University of Eastern Finland, Kuopio, Finland.

### 2.2. Contrast-Enhanced Ultrasound Imaging of Skeletal Muscle Perfusion

The resting blood flow in the calf muscles was monitored and quantitatively measured pre-operatively and at 0, 1, 4, 7, 11 and 29 days after the surgery with the Acuson Sequoia 512 ultrasound system and a 15L8 transducer (Siemens, Malvern, PA, USA) using contrast-enhanced Power Doppler (CED: frequency = 14 MHz, power = −5 dB, Doppler gain = 50) and Cadence contrast pulse sequencing (CEU: frequency = 14 MHz, power = −8 dB, mechanical index = 0.25, CEU gain = 0 and depth = 20 mm) software [14]. CED imaging allows for the measurement of the flux of red blood cells, primarily through high-pressure arteries and large veins, while CEU imaging enables the measurement of real-time tissue perfusion at a microvascular level, with a superior spatial resolution showing blood flow in vessels with a diameter of 10 to 20 µm in addition to bigger vessels [14,15]. After isoflurane anesthesia, both hindlimbs, operated and contralateral, were placed in a prone position over the holder, fixed with tape and immersed in Aquasonic 100 ultrasound transmission gel (Parker Laboratories Inc., Fairfield, NJ, USA). The transducer was placed on top of the calf muscle bundle in a transverse position. Perfusion video clips (20 s in length, 15 clip frames/1 s) were recorded upon the administration of an intravenous bolus injection of 50 μL of SonoVue contrast agent (a sulfur hexafluoride gaseous core in a phospholipid shell, approx. 2 × 10^8^ bubbles/mL, mean size 2.5 µm, Bracco, Milan, Italy) via the jugular vein. The CED and CEU peak signal intensities (dB; relative to both blood flow and volume) of the video clips were quantified with Datapro software (v2.13, Noesis, Courtaboeuf, France). In addition, the contrast arrival time, i.e., the time (in seconds) from the administration of the bolus injection to the arrival of the contrast agent into the imaging plane, was calculated from the CEU video clips as a surrogate of the changes in arterial driving pressure. In cases in which the contrast agent did not arrive at the imaging plane within the 20-second-measurement window, the maximal time was recorded for the arrival time.

### 2.3. Photoacoustic Imaging of Microvascular Hemoglobin Oxygenation

Microvascular hemoglobin oxygenation in calf muscles was monitored and quantitatively measured pre-operatively and at 0, 1, 4, 7, 11 and 29 days after the ischemia operation with the Vevo 2100 LAZR photoacoustic imaging (PAI) system equipped with a linear-array transducer (LZ250, center frequency = 21 MHz, 256 elements; VisualSonics Inc., Toronto, ON, Canada) [16]. The measurements were made in the oxy-hemoglobin quantification mode with the following parameters: frequency = 21 MHz, PA gain = 50 dB, 2D gain = 18 dB, depth = 20 mm, width = 23.04 mm and dual laser wavelength = 750/850 nm. This method has already been used in several applications for non-invasive measurements of microvascular blood oxygenation levels in animals and humans [17,18,19,20,21]. Upon isoflurane anesthesia, both the operated and contralateral hindlimb were positioned in a similar fashion for contrast-enhanced ultrasound imaging and immersed in Aquasonic Clear ultrasound transmission gel (Parker Laboratories Inc., Fairfield, NJ, USA). Upon placement of the transducer across both calves, transverse plane photoacoustic clips, superimposed on B-mode ultrasound clips, were acquired. The average microvascular oxygen saturation of hemoglobin at a muscle level (mHbO_2_%) was measured from at least 30 clip frames using Vevo LAB software (v1.7.2, VisualSonics Inc., Toronto, ON, Canada).

### 2.4. Histological Approaches

After euthanization with carbon dioxide, the mice were perfusion-fixed through the left ventricle with 1% paraformaldehyde. The posterior calf muscle bundle, including the two-headed gastrocnemius, plantaris and soleus muscles, was immersion-fixed in 4% paraformaldehyde in 7.5% sucrose (pH 7.4) for 4 h followed by 15% sucrose (pH 7.4) for at least 12 h. After fixation, the samples were embedded in paraffin and prepared into 4 µm thick transversal sections.

**Figure 1:**
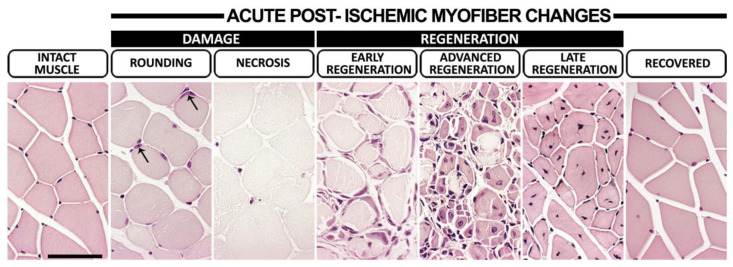
**Myofiber morphologies during post-ischemic damage and regeneration.** Representative hematoxylin–eosin (HE)-stained images displaying intact calf muscle and different stages of morphological changes to myofibers following acute femoral arterial ligation. In intact muscles, eosinophilic myofibers displayed an angular shape with peripherally located condensed, dark nuclei in addition to relatively uniform fiber diameters and organized muscle fascicles. Ischemic myofiber damage was presented either as rounding, i.e., a loss of polygonal shape and a pale eosinophilic sarcoplasmic coloring of myofibers with still preserved muscle fascicle organization, and/or necrosis, i.e., very pale non-nucleated rounded myofibers and a loss of muscle fascicle organization. Arrows indicate hypertrophic non-condensed nuclei of activated satellite cells in the periphery of scattered rounded myofibers. Basophilic satellite cell rings surrounding necrotic myofibers were considered signs of early regeneration. The appearance of small rounded myofibers with multiple hypertrophic non-condensed centralized nuclei together with necrotic myofiber remnants and/or fusing together to form larger units marked advanced regeneration. Angularly shaped eosinophilic myofibers organized in muscle fascicles with centrally oriented, condensed, dark nuclei and significantly varying fiber diameters were considered late regeneration and preceded completely recovered myofiber morphology. Scale bar: 50 µm.

To analyze the ischemia-induced myofiber changes, calf muscle bundle sections were stained with hematoxylin-eosin (HE). Images of the histological sections were taken with an Olympus AX-70 light microscope (Olympus Optical, Tokyo, Japan) at a 1.25× magnification. The entire cross-sectional muscle area was examined using analySIS imaging software (v3.00, Soft Imaging System GmbH, Muenster, Germany), and the following six areas, representing different myofiber morphologies (Figure 1), were measured: (1) normal area; (2) rounded myofibers; (3) necrosis; (4) early regenerative changes; (5) advanced regeneration; (6) late regeneration. Each area was calculated as a percentage of the whole cross-sectional muscle area. The damaged muscle area was calculated as the sum of areas 2–3. The regenerative muscle area was calculated as the sum of areas 4–6.

For the immunohistochemical assessment of post-ischemic muscle capillary reactivity, calf muscle sections were immunostained with antibodies against CD31 (platelet endothelial cell adhesion molecule (PECAM-1), dilution 1:25, rat anti-mouse monoclonal CD31, MEC 13.3; BD BioSciences Pharmingen, San Diego, CA, USA), α-SMA (dilution 1:50, anti-alpha smooth muscle actin-Cy3 mouse monoclonal, clone 1A4; Sigma-Aldrich, Darmstadt, Germany) and Ki-67 (a proliferation marker, dilution 1:500, rabbit polyclonal, ab15580; Abcam, Cambridge, UK). Biotinylated rabbit anti-rat secondary antibody (dilution 1:200, Vector Laboratories, Burlingame, CA, USA) was used for the CD31 and α-SMA stainings, and biotinylated horse anti-rabbit secondary antibody (dilution 1:200, Vector Laboratories, Burlingame, CA, USA) was used for the Ki-67 staining. The avidin-biotin-horseradish peroxidase system (Vector Laboratories, Burlingame, CA, USA) with 3,3’-Diaminobenzidine (DAB) as a chromogen (Zymed, San Francisco, CA, USA) was used to visualize the immunoreactivity for light imaging. Tyramide signal amplification (TSA, PerkinElmer, Shelton, CT, USA) was used to enhance the signal. Calf muscle slides were mounted with Permount (ThermoFisher Scientific, Waltham, MA, USA) for brightfield imaging, or the Vectashield antifade mounting media with DAPI (Vector Laboratories, Burlingame, CA, USA) was used for fluorescent imaging. Fluorescent imaging was performed with a Zeiss LSM700 confocal microscope (Oberkochen, Germany), where 405/488/555 nm diode lasers together with the appropriate emission filters were used (20×/0.5 PlanApo objectives, 1024 × 1024 frame sizes).

### 2.5. Capillary Measurements

To analyze the post-ischemic muscle capillary responses, images of the CD31-stained histological sections were taken with a Nikon H550L light microscope (Tokyo, Japan) and NIS-Elements advanced research imaging software at a 20× magnification. To characterize the types of alterations occurring in the capillary responses over time, only areas with myofiber histopathological changes (if applicable) were selected for the evaluation of CD31-based capillary reactivity, as described before [22]. capillary area (endomysial CD31^+^ mean vessel luminal area in µm^2^), capillary density (endomysial CD31^+^ vessels/muscle area mm^2^) and capillary size distribution (fraction (%) of the endomysial CD31^+^ vessels bigger than an average normal capillary, here defined as 33 µm^2^ based on the counting of 12,326 capillaries from intact muscle sections of C57Bl/6J mice) were measured from two to three fields for each calf muscle using NIS-Elements software (v4.51.01, Nikon, Tokyo, Japan). Calf muscle bundle cross-sections, derived from intact C57Bl/6J mice (*n* = 6) and LDLR^−/−^ApoB^100/100^ mice (*n* = 8), were used to define the baseline for the corresponding mouse strains. All measurements were performed in a blind manner.

### 2.6. Statistical Analysis

All statistical analyses were performed using SPSS software (v27.0 for Windows, SPSS Inc., Chicago, IL, USA) and a *p* < 0.05 was considered statistically significant. Data were expressed as the mean ± standard error of the mean (SEM) with individual data points displayed on top of the plots. Significant changes within each strain were assessed using the Kruskal–Wallis test followed by the Mann–Whitney U test. The difference in the prevalence of necrosis between the two mouse strains was assessed using the Chi-squared test.

## 3. Results

### 3.1. The “Favorable” and “Delayed” Patterns of Ischemic Damage and Regeneration

**Figure 2:**
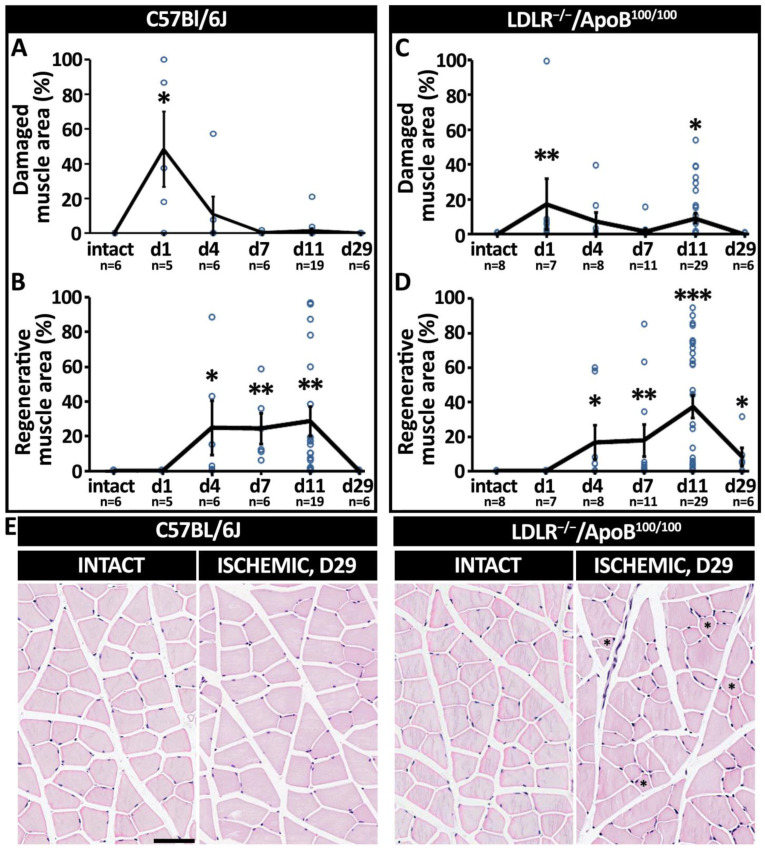
**Aged, healthy C57Bl/6J and aged, hyperlipidemic LDLR^−/−^ApoB^100/100^ mice displayed differential patterns of post-ischemic damage and regeneration**. HE-based quantitation of (**A**) myofiber damage and (**B**) regeneration in aged, healthy C57Bl/6J mice. HE-based quantitation of (**C**) myofiber damage and (**D**) regeneration in aged, hyperlipidemic LDLR^−/−^ApoB^100/100^ mice. (**E**) Post-ischemic recovery led to complete normalization of myofiber morphology in C57Bl/6J but not in LDLR^−/−^ApoB^100/100^, where, on top of the still late regenerative changes (**D**) myofiber atrophy (asterisks) was also detected on day 29, as shown in the HE staining. * *p* < 0.05, ** *p* < 0.01, *** *p* < 0.001 vs. intact muscle. Scale bar: 50 µm.

Following the acute ischemia operation, the presentation of macroscopic signs of ischemia was most commonly seen in the C57Bl/6J mice on day 1 but in LDLR^−/−^/ApoB^100/100^ mice on day 4. The most prevalent macroscopic symptoms were swelling and/or stiffness of the operated hindlimbs, which were observed in 18% and 39% of C57Bl/6J and LDLR^−/−^/ApoB^100/100^ mice, respectively. More severe symptoms, such as toe/toenail necrosis (2% in C57Bl/6J and 28% in LDLR^−/−^/ApoB^100/100^, Chi-squared, *p* < 0.001) or toe auto-amputation (5% in both strains) were less common and rare, respectively. Histologically, the myofiber responses to acute ischemia in the C57Bl/6J mice were presented in two phases: an initial phase of ischemic damage followed by a phase of regeneration. The HE analysis of the skeletal muscles (Figure 1) revealed that, on day 1, on average, 48% of the calf muscle in the C57Bl/6J mice was acutely injured by the operation (*p* < 0.05; Figure 2A). On day 4, on average, 11% of the total muscle area was displaying necrosis (Figure 2A). The regenerative changes (Figure 1) were first presented on day 4, persisted at least until day 11 (Figure 2B) and led to the complete recovery of the myofiber morphology by day 29 in the C57Bl/6J mice (Figure 1). In the LDLR^−/−^/ApoB^100/100^ mice, the ischemic muscle damage already presented two phases. Acute damage on day 1 was detected on average in 17% of the calf muscle area, whereas a second peak of damage occurred on day 11, affecting, on average, 9% of the calf muscle (*p* < 0.05; Figure 2C). Hallmarks of early regeneration (Figure 1) in the LDLR^−/−^/ApoB^100/100^ mice were observed as early as day 4 but persisted until day 11, leaving the calf muscles still under repair on day 29 (*p* < 0.05; Figure 2D). Unlike C57Bl/6J, the LDLR^−/−^/ApoB^100/100^ muscles on day 29 also showed atrophic, non-regenerating myofibers, indicating possible chronic damage resulting from the acute ischemia under hyperlipidemic conditions (Figure 2E). The ischemic damage and regeneration in the LDLR^−/−^/ApoB^100/100^ mice appeared therefore delayed, whereas the C57Bl/6J mice displayed a more favorable swift recovery, regardless of old age.

### 3.2. Skeletal Muscle Blood Flow Recovers under Decreased Arterial Driving Pressure

**Figure 3:**
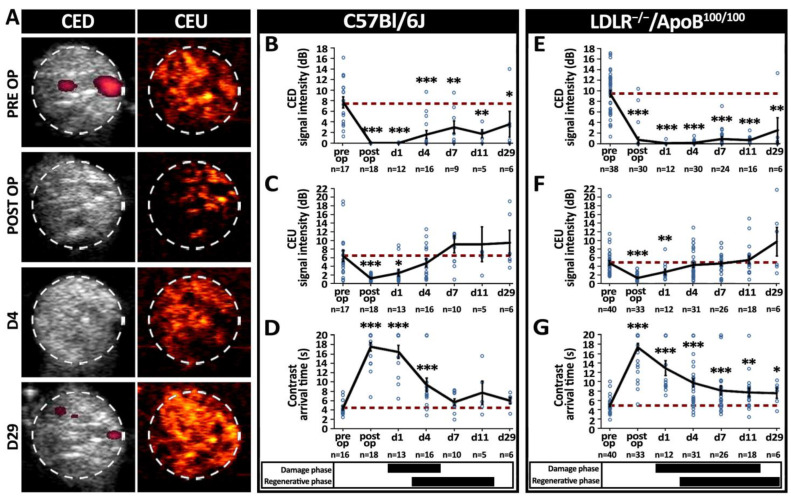
**Post-ischemic restoration of microvascular blood flow took place under decreased arterial driving pressure in both strains, but arterial driving pressure further recovered only in C57Bl/6J mice**. (**A**) Contrast-enhanced Power Doppler (CED) and contrast-enhanced ultrasound (CEU) were used to detect macro and microvascular blood flow recovery, respectively. White dash circles display the analysis area of a single-leg cross-section in each image. Quantitation of changes in (**B**) macrovascular blood flow using CED, (**C**) microvascular blood flow using CEU and (**D**) CEU contrast arrival time, reflecting arterial driving pressure in aged, healthy C57Bl/6J showing a recovery of tissue-level microvascular blood flow on day 4, a recovery of arterial driving pressure on day 7 but still significantly reduced CED signal on day 29. Quantitation of changes in (**E**) macrovascular blood flow using CED, (**F**) microvascular blood flow using CEU and (**G**) CEU contrast arrival time, reflecting arterial driving pressure in aged, hyperlipidemic LDLR^−/−^ApoB^100/100^ showing a recovery of tissue-level microvascular blood flow on day 4 but still significantly altered CED signal and contrast arrival time on day 29. Red dash line in (**B**–**G**) indicates the baseline level. * *p* < 0.05, ** *p* < 0.01, *** *p* < 0.001 vs. pre op values. The timing of phases of histological damage and regeneration (from Figure 2) are displayed under the graphs (**B**–**G**) of the corresponding mouse strain.

CED and high-resolution CEU ultrasound were used to differentiate between arterial/venous and microvascular blood flow, respectively (Figure 3A). Whereas both methods uniformly displayed a significant reduction in blood flow after ischemia operation, the level of postoperative flow and the detection of flow recovery differed between the methods. In C57Bl/6J mice, the CED showed no significant recovery of large arterial/venous flow in the operated hindlimbs throughout the follow-up (Figure 3B). Instead, high-resolution CEU imaging of the C57Bl/6J mice showed some contrast signal appearance immediately after the operation and recovery of microvascular flow to the pre-operative values as early as 4 days after the operation (Figure 3C). Importantly, the CEU contrast arrival time in the C57Bl/6J mice recovered back to the baseline levels only 7 days post-operation, reflecting the recovery of arterial driving pressure (Figure 3D). Also, in LDLR^−/−^/ApoB^100/100^ mice, the CED flow remained significantly reduced throughout the 29-day follow-up (Figure 3E), and the CEU contrast signal was restored to baseline levels 4 days after the operation (Figure 3F). However, the CEU contrast arrival time in the LDLR^−/−^/ApoB^100/100^ mice remained significantly reduced throughout the whole 29-day follow-up (Figure 3G). The recovery of skeletal muscle blood flow on the level of the microvasculature, therefore, seems to take place under decreased arterial driving pressure and in the absence of large arterial flow in both studied mouse strains. Arterial driving pressure was recovered in C57Bl/6J but not in LDLR^−/−^/ApoB^100/100^ mice, based on the CEU contrast arrival time.

### 3.3. Oxygen Delivery and Its Tissue Demand Affect Post-Ischemic Microvascular Hemoglobin Oxygenation

**Figure 4:**
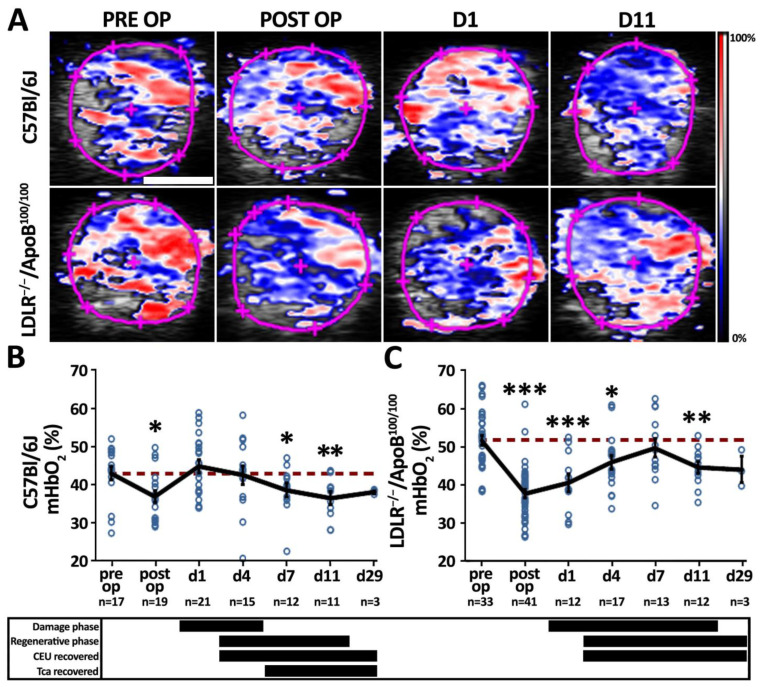
**Normalization of microvascular hemoglobin oxygenation happened unpredictably fast in C57Bl/6J mice but decreased again after normalization in both mouse strains**. (**A**) Photoacoustic imaging (PAI) was used to non-invasively study changes in microvascular hemoglobin oxygenation (mHbO_2_%). Pink circles on the images represent the analysis area of a single-leg cross-section in each image. Scale bar: 2 mm. (**B**) PAI-based quantitation of mHbO_2_% in aged, healthy C57Bl/6J displays an initial normalization on day 1 followed by a significant decrease on days 7–11. (**C**) Normalization of mHbO_2_% in aged, hyperlipidemic LDLR^−/−^ApoB^100/100^ takes place on day 7, after which mHbO_2_% decreases again on day 11. Red dash line in (**B**,**C**) indicates the baseline level. * *p* < 0.05, ** *p* < 0.01, *** *p* < 0.001 vs. pre op values. The timing of phases of histological damage and regeneration (from Figure 2), and recovery of microvascular CEU blood flow and contrast arrival time (Tca) (from Figure 3) are displayed under the graphs (**B**,**C**) of the corresponding mouse strain.

To assess the functionality of the tissue microcirculation after ischemic injury, PAI was used to measure alterations in the microvascular hemoglobin oxygen saturation (mHbO_2_%) in the affected calf muscles (Figure 4A). Immediately after the operation, the mHbO_2_% in the operated hindlimbs significantly decreased from the pre-operative values as expected, reflecting the suddenly reduced arterial inflow of oxygenated blood and increased oxygen extraction from hemoglobin in the acutely ischemic muscles. However, only hours after the operation, the mHbO_2_% in individual C57Bl/6J mice reached baseline values (Figure 4A), leading to the normalization of a mean mHbO_2_% already by day 1 (Figure 4B). Interestingly, the normalized mHbO2% in C57Bl/6J mice was maintained only until day 4, after which a second significant decrease in mHbO_2_% was detected on days 7 and 11. The mHbO_2_% recovery in LDLR^−/−^/ApoB^100/100^ mice took place only on day 7 and thereafter, again significantly decreased 11 days post-surgery (Figure 4C). In both strains, the second reduction in the mHbO_2_% seemed to take place in the presence of a fully restored microvascular blood flow, suggesting increased oxygen demand of the tissue rather than reduced arterial delivery of oxygenated blood. However, the recovery of the mHbO_2_% under still significantly reduced muscle blood flow in C57Bl/6J mice on day 1 could suggest an impairment of oxygen extraction in severely hypoxic muscles.

### 3.4. Initial Capillary Enlargement Launches a Cascade of Post-Ischemic Microvascular Changes That Lead to Increased Capillary Density in C57Bl/6J but Not in LDLR^−/−^/ApoB^100/100^ Mice

**Figure 5:**
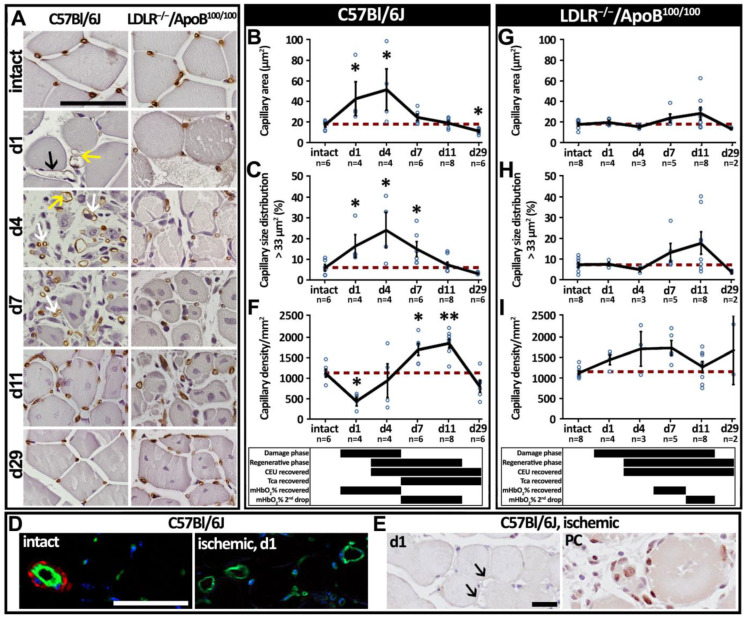
**Initial capillary enlargement was followed by an increase in capillary density in the C57Bl/6J, whereas the LDLR^−/−^ApoB^100/100^ displayed no significant capillary changes.** (**A**) Representative CD31 (brown) immunohistochemical stainings displaying the morphological appearance of skeletal muscle capillaries in aged, healthy C57Bl/6J and aged, hyperlipidemic LDLR^−/−^ApoB^100/100^ at different time points. Appearance of thin-walled, enlarged capillaries (black arrow) was detected on day 1 in C57Bl/6J. These vessels often displayed CD31^+^ intraluminal pillar-like structures (yellow arrows). On days 4–7, small, paired capillaries were found next to each other (white arrows) in C57Bl/6J, indicating possible daughter vessels formed through intussusceptive angiogenesis. CD31-based quantitation of (**B**) capillary area and (**C**) capillary size distribution as well as (**D**) immunofluorescence staining with CD31 (green), α-sma (red) and DAPI (blue), and (**E**) Ki-67 staining demonstrated that the capillaries are enlarged, lack α-sma-positive smooth muscle cells and do not display endothelial proliferation (black arrows) in C57Bl/6J on days 1–4. This was followed by (**F**) an increase in capillary density on days 7–11. CD31-based quantitation of (**G**) capillary area, (**H**) capillary size distribution and (**I**) capillary density showed no significant differences in LDLR^−/−^ApoB^100/100^. Red dash line in (**B**,**C**,**F**–**I**) indicates the baseline level. * *p* < 0.05, ** *p* < 0.01 vs. intact. The timing of phases of histological damage and regeneration (from Figure 2), recovery of microvascular CEU blood flow and contrast arrival time (Tca) (from Figure 3), and hemoglobin oxygenation recovery (mHbO_2_% recovered) and hemoglobin oxygenation second decrease (mHbO_2_% 2nd drop) (from Figure 4) are displayed below the graphs (**B**,**C**,**F**–**I**) of the corresponding mouse strain. Scale bars in (**A**,**D**): 50 µm. Scale bar in (**E**): 20 µm. PC—positive control.

CD31 immunostainings were used to analyze the responses of the muscle capillary bed to acute ischemia (Figure 5A). In C57Bl/6J mice, capillaries in areas of muscle damage on days 1–4 post-operation were significantly enlarged when compared to the intact muscle (*p* < 0.05; Figure 5B). Furthermore, especially on day 1, C57Bl/6J capillaries displayed relatively thin capillary walls as compared to the capillaries in healthy skeletal muscle (Figure 5A, black arrow). capillary size distribution, representing the fraction of capillaries undergoing significant enlargement, demonstrated that as much as 24% of the endomysial blood vessels in ischemic muscles of C57Bl/6J mice had an increased luminal area on day 4 (*p* < 0.05; Figure 5C). The enlarged endomysial blood vessels in C57Bl/6J on day 1 also displayed no α-SMA-smooth muscle cell coverage, observed by immunofluorescence (Figure 5D, ischemic). In contrast, vessels of a similar caliber in the perimysial space of the intact muscles (small arterioles or venules) were α-SMA-positive (Figure 5D, intact). Enlarged capillaries of the C57Bl/6J mice on day 1 were also not displaying endothelial cell proliferation, as assessed by Ki-67 immunostaining (Figure 5E, d1, black arrows). Instead, nuclei of the proliferating satellite cells on day 11 were Ki67-positive (Figure 5E, positive control (PC)). During the period of capillary enlargement, capillary density in C57Bl/6J mice was initially decreased (*p* < 0.05; Figure 5F) but quickly normalized and further significantly increased along normalization of capillary size on days 7 to 11 (*p* < 0.05; Figure 5F). Possibly mediating this transition, CD31-positive endothelial pillars, resembling vascular structures in intussusceptive angiogenesis, were detected crossing lumens of enlarged capillaries on days 1 and 4 (Figure 5A, yellow arrows) and possible daughter vessels, which were located in very close proximity to each other on days 4 and 7 (Figure 5A, white arrows). Capillary dilation, therefore, appears as the first response of the tissue capillary bed to ischemia, leading to further normalization of capillary size and an increase in capillary density that seems to mediate tissue recovery in C57Bl/6J mice. Capillaries in LDLR^−/−^/ApoB^100/100^ mice, neither in the areas of muscle damage nor in the areas of regeneration at any studied time point, showed significant changes in the mean capillary area (Figure 5G), capillary size distribution (Figure 5H) and capillary density (Figure 5I).

## 4. Discussion

Previous studies have extensively addressed the mechanisms through which hyperlipidemia can impair endothelial function and arterial smooth muscle relaxation [23,24,25,26,27]. This study continues from those findings and demonstrates how hyperlipidemia blunts microvascular angiogenesis and, through the impairment of capillary function, alters differential physiological parameters compromising post-ischemic muscle recovery. This study highlights the importance of proper capillary endothelial function in post-ischemic recovery. A sequence of dynamic capillary responses was associated with successful post-ischemic muscle repair in aged but otherwise healthy C57Bl/6J mice. Instead, the lack of capillary responses in aged, hyperlipidemic LDLR^−/−^/ApoB^100/100^ mice was associated with a delay in overall post-ischemic recovery. Beyond muscle regeneration, the differential capillary responses in the two mouse strains were also linked to changes in the blood flow parameters, microvascular hemoglobin oxygenation, as well as the extent of ischemic damage.

Capillary enlargement, identified here as the primary post-ischemic capillary response (on days 1 to 4) in C57Bl/6J mice, affected only about 20% of the capillaries in the capillary bed. The quick selective enlargement of capillaries could suggest the involvement of physical forces, such as collateral flow distribution, in initiating this process and, as such, may be dependent on efficient arterial smooth muscle relaxation [23,27,28]. In support of flow-mediated capillary vasodilation, the walls of enlarged capillaries in the C57Bl/6J mice were relatively thin on day 1 and did not show Ki67-positivity as a sign of endothelial cell proliferation. Through decreasing peripheral resistance, flow-mediated post-ischemic capillary enlargement could also facilitate collateral maturation. Enlarged capillaries allow high flow through the microvascular bed [29], which in turn can be expected to help the maturation of upstream nascent collaterals into high-pressure-enduring functional conduits through, e.g., shear stress-mediated mechanisms [2,30]. To support this conclusion, the arterial arrival time of the CEU contrast agent was recovered in C57Bl/6J but not in LDLR^−/−^/ApoB^100/100^ mice. Based on CEU imaging, the gradually decreasing but still significantly slower arrival of the contrast agent on day 4 suggests that blood flow to the ischemic calf muscles in both strains was initially reinstated via relatively narrow collateral arteries with steadily increasing arterial driving pressure. The normalization of the contrast arrival time on day 7 in C57Bl/6J mice could imply further growth and maturation of collaterals to the point of normalizing arterial driving pressure. Notably, upon the restoration of arterial driving pressure, the effect of significant capillary enlargement also disappeared as the capillary size distribution returned to normal in C57Bl/6J mice. Instead, the lack of capillary enlargement may implicate impaired collateral remodeling in LDLR^−/−^/ApoB^100/100^ mice, resulting in an inability to retrieve perfusion pressure, as indicated by the significantly delayed contrast arrival time still on day 29. Interestingly, the lack of capillary enlargement in LDLR^−/−^/ApoB^100/100^ mice did not seem to affect the initial opening of collaterals, as the return of tissue blood flow took place on day 4, which was similar to the C57Bl/6J mice. Initial post-ischemic capillary enlargement, therefore, seems an important initiator of post-ischemic capillary remodeling that could facilitate collateral maturation and the normalization of arterial driving pressure rather than the initial recovery of tissue blood flow.

Capillary enlargement was also associated with paradoxically increased microvascular hemoglobin oxygenation in the acutely ischemic C57Bl/6J muscles. Computational modeling has predicted that the rate of oxygen diffusion from the blood of the capillaries to myofiber mitochondria might be accelerated through a network of cell membrane phospholipids [31]. The small diameter of capillaries, therefore, could serve to maximize the contact area between the cell membrane phospholipids of erythrocytes and capillary endothelium for the optimal diffusion of oxygen to adjacent myofibers. Capillary enlargement, detected in C57Bl/6J mice on day 1, could have reduced the contact area between the red blood cells and capillary endothelium. According to Pias, this might paradoxically compromise oxygen delivery through enlarged capillaries and further exacerbate acute muscle damage. In support of this theory, in this study, the normalization of hemoglobin oxygenation was detected in the acutely ischemic muscles without fully recovered microvascular blood flow in C57Bl/6J mice. In contrast, the absence of initial capillary enlargement in the LDLR^−/−^/ApoB^100/100^ mice did not seem to hamper oxygen extraction postoperatively, as the mHbO_2_% remained significantly decreased on days 1 to 4. However, a trend towards an increase in the fraction of capillaries undergoing some degree of enlargement in the LDLR^−/−^/ApoB^100/100^ mice was observed on days 7 to 11. This coincided with the normalization of the mHbO_2_% in LDLR^−/−^/ApoB^100/100^ mice on day 7 and with the time of the second significant peak of muscle damage observed in LDLR^−/−^/ApoB^100/100^ in the presence of fully recovered microvascular flow on day 11. Hence, capillary enlargement seems to be able to affect oxygen diffusion from erythrocytes to tissues and potentially could also cause direct tissue damage.

Interestingly, in this study, the degree and timing of the post-ischemic damage seen in the two mouse strains did not relate to the amount of blood flow but rather to the extent of capillary enlargement and its impact on arterial driving pressure return. Despite the equivalent reduction of postoperative blood flow in both strains, macroscopic signs of ischemic damage, such as swelling of the leg, were most common in C57Bl/6J on day 1, whereas they were most prevalent in LDLR^−/−^/ApoB^100/100^ only on day 4. Tissue edema is commonly associated with angiogenesis and capillary enlargement [29] and could also explain the differential macroscopic findings here. The presence of transient capillary enlargement in C57Bl/6J mice may have also protected them from distal necrosis. Irrespective of a similar recovery of postoperative microvascular blood flow on day 4, 28% of LDLR^−/−^/ApoB^100/100^ and only 2% of C57Bl/6J mice showed distal necrosis. This may be explained by the insufficient arterial driving pressure in LDLR^−/−^/ApoB^100/100^ not being able to mediate enough blood to the most distal parts of the limb. The insufficient arterial driving pressure in LDLR^−/−^/ApoB^100/100^ likely also contributed to the formation of chronic damage in the ischemic muscles. The role of capillary enlargement in post-ischemic recovery may thus be controversial. The initial phase of capillary vasodilation might increase acute tissue damage but could protect from more chronic damage by facilitating collateral maturation and the recovery of arterial driving pressure.

The phase of capillary enlargement rapidly ended in C57Bl/6J mice, along with the normalization of arterial driving pressure on day 7. After this, a period of increased capillary density (days 7 to 11) took over and displayed abundant myofiber regeneration. This clear switch of capillary response points towards the possible involvement of intussusceptive angiogenesis in expanding the capillary network. Increased blood flow has been previously found to initiate the intussusceptive splitting of enlarged capillaries [32,33]. As compared to sprouting angiogenesis, intussusception has also been reported to be both faster and more energy efficient [34], providing a possible benefit in ischemic tissues to expand the capillary network according to the needs of the regenerating muscle. Pointing towards intussusception, CD31-positive intraluminal pillars were found in enlarged capillaries on days 1 to 4. Later, on days 4 to 7, small “paired” capillaries were often spotted, representing possible split daughter vessels. Supportive evidence of intussusceptive angiogenesis, being responsible for the regeneration of microvasculature in ischemic skeletal muscle, was also described in a recently published study by Arpino et al. [35]. The phase of increased capillary density in C57Bl/6J mice was also associated with another decrease in hemoglobin oxygenation during already recovered microvascular blood flow and arterial driving pressure. It is likely that this decrease in mHbO_2_% was induced by the energy-consuming process of satellite cell-induced muscle regeneration [36]. Adequate functional perfusion and optimal oxygen diffusion through small-sized capillaries are likely preset for efficient regeneration. Activated satellite cells have been previously reported to proliferate near capillaries [6,7] and to be actively involved in recruiting endothelial cells through VEGF signaling [7]. Similarly, in C57Bl/6J mice, hallmarks of advanced regeneration, such as proliferation, maturation and fusion of satellite cells, were exclusively seen in areas with increased capillary density, leading to a complete morphological recovery on day 29. Despite the impaired capillary responses, LDLR^−/−^/ApoB^100/100^ muscles also displayed regeneration. However, the regenerative process in LDLR^−/−^/ApoB^100/100^ was delayed and even revealed signs of chronic damage, such as myofiber atrophy, on day 29.

## 5. Conclusions

Based on these results, the activation of a “favorable” capillary response is required for successful post-ischemic tissue recovery. That is, (1) an early capillary dilation phase that facilitates the return of arterial driving pressure prior to (2) an intussusceptive increase in capillary density, supporting efficient oxygen delivery and muscle regeneration. Failure of this capillary response, again, leads to an inability to recover arterial driving pressure, resulting in an increase in distal necrosis, induction of chronic damage and slowing down of myofiber regeneration. In conclusion, the microvasculature reveals an important role in regulating post-ischemic muscle recovery. Understanding the dynamic nature of the post-ischemic capillary bed is essential for designing novel therapies that target the microvasculature in ischemic diseases.

## Data Availability

The datasets used and/or analyzed during the current study are available from the corresponding author on reasonable request.
